# Molecular regulators of alcoholic liver disease: a comprehensive analysis of microRNAs and long non-coding RNAs

**DOI:** 10.3389/fmed.2025.1482089

**Published:** 2025-03-10

**Authors:** Lin Zhang, Rongqi Wang, Yuemin Nan, Lingbo Kong

**Affiliations:** Department of Traditional and Western Medical Hepatology, Hebei Medical University Third Hospital, Shijiazhuang, China

**Keywords:** alcoholic liver disease, MicroRNAs, long non-coding RNAs, gene regulation, therapeutic targets

## Abstract

Many biomolecules and signaling pathways are involved in the development of alcoholic liver disease (ALD). The molecular mechanisms of ALD are not fully understood and there is no effective treatment. Numerous studies have demonstrated the critical role of non-coding RNAs, including long non-coding RNAs (lncRNAs) and microRNAs (miRNAs), in ALD. miRNAs play an important regulatory role in the pathogenesis of ALD by controlling critical biological processes such as inflammation, oxidative stress, lipid metabolism, apoptosis and fibrosis. Among them, miR-155, miR-223 and miR-34a play a central role in these processes and influence the pathological process of ALD. In addition, lncRNAs are involved in regulating liver injury and repair by interacting with miRNAs to form a complex regulatory network. These findings help to elucidate the molecular mechanisms of ALD and provide a scientific basis for the development of new diagnostic markers and therapeutic targets. In this article, we review the roles and mechanisms of LncRNAs and miRNAs in ALD and their potential use as diagnostic markers and therapeutic targets.

## 1 Introduction

Alcoholic liver disease (ALD) is a group of liver injuries and diseases caused by chronic excessive alcohol consumption (1). With the improvement in living standards and lifestyle changes in society, alcohol consumption has increased, leading to a yearly increase in the incidence of alcoholic liver disease, which has become a major global public health challenge (2). The pathophysiological process of ALD is very complex and includes different stages such as fatty liver, alcoholic hepatitis (AH) and alcoholic cirrhosis (3). In the early stages, alcohol causes fat to accumulate in the liver, resulting in fatty liver. Over time, secondary inflammation and fibrosis can lead to AH, which can eventually lead to serious consequences such as cirrhosis and hepatocellular carcinoma. This process involves the complex regulation of multiple cellular and molecular mechanisms such as apoptosis, oxidative stress and the release of inflammatory mediators (4). In addition to direct damage to the liver, ALD is often associated with a number of systemic complications, including malnutrition (e.g., vitamin deficiency), neurological damage (e.g., hepatic encephalopathy, peripheral neuropathy), cardiovascular disease (e.g., alcoholic cardiomyopathy), and immune dysfunction (5). For example, miR-155 was found to exacerbate cardiovascular injury by upregulating systemic inflammatory factors (e. g., TNF- *α*, IL-6) release (6). The miR-34a promotes hepatocyte apoptosis through the SIRT 1 / p53 pathway, and its released exosomes may affect the CNS (7, 8). lncRNA CRNDE By activating inflammatory factors such as IL-6, it may mediate multiorgan inflammatory (9, 10) through circulating exosomes.Therefore, in-depth research and effective intervention in ALD are of great importance.

Most (76–97%) of the human genome encodes RNAs that are not translated into proteins, called non-coding RNAs (ncRNAs). NcRNAs are functional RNA molecules that are not directly involved in translation and play an important role in fine-tuning cellular functions. Based on the difference in length, ncRNAs can be divided into short and long ncRNAs. Among them, miRNAs are a specific class of short ncRNAs, approximately 22 nucleotides in length. miRNAs achieve degradation or inhibition of mRNA translation by complementary pairing with the 3' untranslated region (3'UTR) of the target mRNA, and thus play an important role in regulating gene expression at the level of gene expression. Specifically, miRNAs inhibit the conversion of mRNAs into proteins by two main mechanisms: degradation of mRNAs and inhibition of translation initiation (11). lncRNAs are more diverse and, like miRNAs, have specific functions depending on their cellular localization (12). Nuclear-localized lncRNAs act as transcription factors or chromatin-modifying complexes (13), and cytoplasmic LncRNAs can directly regulate mRNA stabilization or act as endogenous competing RNAs that regulate functional proteins (14). Most lncRNAs are transcribed by RNA polymerase II or other RNA polymerases and undergo a splicing process similar to that of mRNAs, with 5-co-terminal capping [7-methyl ouabain (m7G)] and 3 m-terminal polyadenylation. lncRNAs have a wide range of biological functions. They are involved in the regulation of gene expression, including DNA replication and transcription, protein translation, and epigenetic lncRNAs are involved in a wide range of biological roles in the regulation of gene expression, including DNA replication and transcription, protein translation, and epigenetic regulation (15). lncRNAs mediate epigenetic modification of DNA by recruiting chromatin remodeling complexes to specific sites (16, 17).

In exploring the interactions between lncRNAs and miRNAs, numerous studies have found that they are jointly involved in post-transcriptional regulatory processes and significantly impact critical aspects of disease onset, metastatic progression and drug resistance (18). By revealing the potential association between lncRNAs and miRNAs, we can gain a deeper understanding of the biological functions of these two RNA molecules and their roles in the pathogenesis of complex diseases (Table 1). This study not only helps us to deepen our understanding of RNA regulatory networks but may also provide new strategies and directions for disease treatment.

**Table 1:** The mechanisms of lncRNA, miRNA action in ALD.

NcRNAs	Expression	Mechanism of action	Target gene
miR-223	UP	Reduced ROS generation	IL-6/p47phox
miR-214	-	Inhibition of GSR and POR expression and activity and promotion of oxidative stress	GSR/POR
miR-181b-5p	UP	Oxidative stress	STAT1
miR-155	UP	Enhancement of kupffer cell responsiveness to LPS Lipid metabolism	TLR4/TNF-α/LPSHDAC11 PPAR
miR-203	Down	Direct inhibition of the downstream target Lipin1 reduces SREBP1expression, inhibits lipid synthesis and promotes fatty acid oxidation	Lipin1/SREBP1
miR-378b	UP	Inhibits CaMKK2,which in turn inhibits the activation of AMPK and promotes the expression of genes related to lipid synthesis	CaMKK2/AMPK/SREBP1
miR-200c	Down	Inhibition of HNF1B and ApoO leads to reduced TG secretion and fatty acid oxidation	HNF1B/ApoO
miR-219a-5p	Down	Inhibition of p66shc expression,which in turn reduces ROS generation	p66shc
miR-let-7a	Down	Promote fibrosis	Lin28B
miR-19b	Down	-	pri-miR-17-92
miR-181b-3p	Down	Inhibition of inflammation	NF-kB
miR-125a-5p	-	Reduced tolerance of kupffer cells to LPS	TLR4/TNF-α/LPSCD14IRAK1
miR-21	UP	Promotes the release of inflammatory factors	VH/NF-kB
miR-182	UP	Directly affects inflammatory mediator expression	-
miR-200a	UP	Promote apoptosis	ZEB2
miR-150-5p	UP	Induction of exogenous apoptosis	CISH/FADD/procaspase-8
miR-34a	- UP	Regulates the SIRT 1-p53 acetylation pathway and promote apoptosis Promote fibrosis	SIRT1 TGF-β1/Smad2/3
miR-148a-3p	Down	Promotes the expression of α-SMA,type I collagen, leading to liver fibrosis	ERBB3
LncRNA AIRN	UP	Inbits mitophagy and promotes hepatocyte injury	mTOR
LncRNA CRNDE	UP	Promote apoptosis Activation of inflammatory factors	MAPK -
LncRNA LINC01093	Down	Targeted inhibition of ICAM-1 and NF-κB signaling pathway increases hepatocyte viability and decreases apoptosis	ICAM-1/NF-κB

In this paper, we review the research progress of lncRNAs and miRNAs in ALD and describe the biological functions and therapeutic potential of lncRNAs, miRNAs and their interactions in the disease.

## 2 Role of miRNAs in ALD

**Figure 1:**
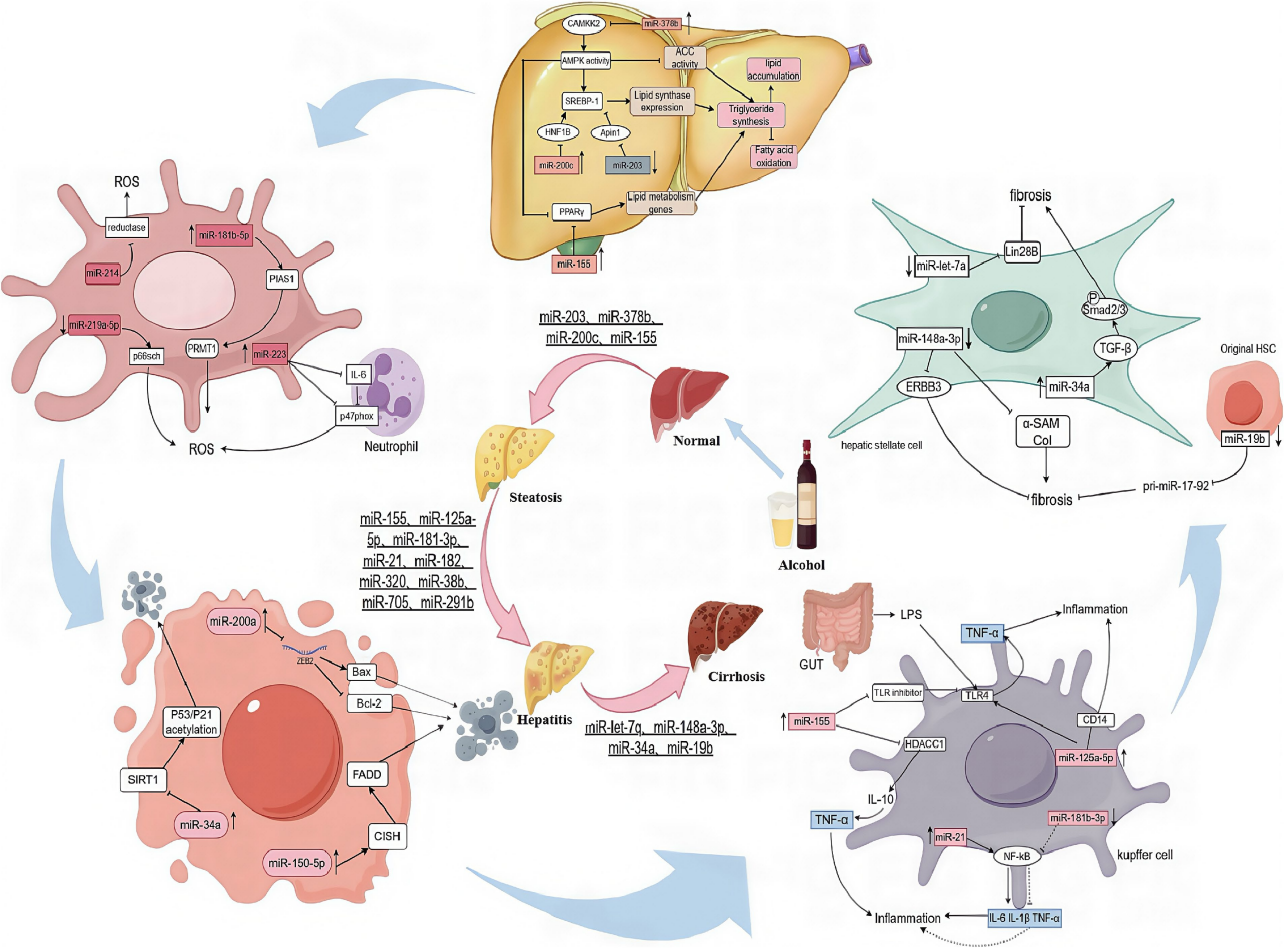
The mechanisms of miRNAs action in steatosis, inflammatory, fibrosis, apoptosis, and oxidative stress.

In recent years, an increasing number of studies have shown that miRNA play a crucial regulatory role in the pathological process of many liver diseases. During the onset and development of ALD, miRNA are not only involved in the regulation of critical biological processes such as hepatocyte steatosis, inflammatory response, fibrosis and apoptosis, but also interact with other ncRNAs such as lncRNAs through a complex molecular network that further influences the disease process. Alcohol-induced oxidative stress and inflammatory response are key aspects of the pathological mechanisms of ALD, and miRNA play an important role in this process by regulating the expression of relevant signaling pathways and target genes. For example, certain miRNA can directly target and regulate genes involved in adipogenesis, inflammatory response, and fibrosis, thereby influencing pathological changes in the liver (19). In addition, alcohol consumption can further exacerbate the condition by affecting the expression profile of miRNA and altering the physiological state of liver cells. This section will review the biological functions of miRNA in ALD and their specific mechanisms, including their roles in steatosis, inflammatory response, fibrosis, and hepatocyte apoptosis (Figure 1).

### 2.1 Lipid metabolism-related miRNA

miRNA play a key role in lipid metabolism in ALD. Sterol Regulatory Element Binding Protein 1 (SREBP1), a major transcriptional regulator of lipid synthesis, is involved in lipid synthesis. miR-203 was able to directly inhibit the downstream target Lipin1, which in turn reduced SREBP1 expression, thereby inhibiting the dual functions of SREBP1 in promoting lipid synthesis and inhibiting fatty acid oxidation. However, in ALD, miR-203 was significantly down-regulated, leading to the over-expression of Lipin1, resulting in lipid over-accumulation and promoting disease progression. miR-203 reduced intracellular lipid synthesis in hepatocytes by targeting and downregulating Lipin1, suggesting that miR-203 is a specific target for the treatment of alcoholic fatty liver disease (19). miR-378b inhibits CaMKK2, which in turn inhibits the activation of AMPK, a key enzyme in the regulation of lipid metabolism that controls the process of lipid metabolism by phosphorylating downstream targets such as acetyl coenzyme A carboxylase ACC. AMPK can phosphorylate SREBP1, inhibiting its activity and repressing the expression of genes involved in its biosynthesis. Another study found that activation of AMPK can affect the protein stability of the peroxisome proliferator-activated receptor *γ* (PPARγ), which is particularly important in lipid metabolism, regulating fatty acid uptake and storage, lipoprotein synthesis and cholesterol metabolism (20). Reduced expression of AMPK leads to an imbalance in the regulation of lipid metabolism, with increased fatty acid synthesis and decreased fatty acid oxidation, exacerbating lipid accumulation in the liver. Inhibition of miR-378b can effectively alleviate ethanol-induced lipid metabolism dysfunction (21). miR-200c directly targets HNF1B (HNF1 homology box B) and ApoO (apolipoprotein O) to regulate lipid homeostasis. These two genes play important roles in lipid metabolism. The expression of miR-200c is significantly upregulated under chronic ethanol exposure. miR-200c contributes to the development of AFL by inhibiting HNF1B and ApoO, leading to reduced TG secretion and decreased fatty acid oxidation, which exacerbates lipid accumulation in the liver (22). Bala S et al. analyzed the critical role of miR-155 in the progression of alcohol-induced liver disease. miR-155 can directly target PPARγ to promote the formation and function of mature adipocytes by activating the expression of lipid metabolism genes such as ACC1 (acetyl CoA carboxylase), FABP4 (fatty acid binding protein), LDLR (low-density lipoprotein receptor-associated protein) and LXR (hepatic X receptor), which increase fatty acid uptake and storage in adipose tissue. By knocking down miR-155, mice were able to significantly resist alcohol-induced steatosis and inflammation, while reducing the degree of alcohol-induced fibrosis. This finding provides strong evidence that miR-155 plays a driving role in promoting alcohol-induced steatohepatitis and fibrosis (6).

### 2.2 Oxidative stress-associated miRNA

miRNA also play an important role in alcohol-associated oxidative stress. miR-219a-5p specifically binds to and represses the expression of p66shc, which causes oxidative cellular damage by regulating ROS (reactive oxygen species) formation in alcoholic liver injury. By inhibiting the expression of p66shc, miR-219a-5p in turn reduces ROS production, thereby alleviating oxidative stress and ROS-induced cellular damage (23). miR-181b-5p indirectly promotes PRMT1 activity by targeting and inhibiting PIAS1 expression, thereby exacerbating oxidative stress and inflammatory responses in AFLD (24). p47phox is a key molecule in the response to oxidative stress and its main function is to stimulate the cell when it receives a stimulus, leading to the activation of NADPH oxidase, the assembly of the enzyme complex and the production of ROS. miR-223 reduces the activation of NADPH oxidase and thus ROS production in neutrophils by directly inhibiting the expression of IL-6 in neutrophils, which in turn inhibits the downstream expression of p47phox. miR-223 is a key molecule in the oxidative stress response (25). Alcohol also induces the expression of miR-214 in hepatocytes. miR-214 reduces ROS scavenging by targeting and inhibiting the expression of genes encoding certain antioxidant enzymes, such as superoxide dismutase (SOD) and glutathione peroxidase (GPx). During alcohol-induced oxidative stress, miR-214 was found to further inhibit the expression and activity of GSR and POR by specifically binding to the 3' untranslated region (3'UTR) of glutathione reductase (GSR) and cytochrome P450 oxidoreductase (POR). By down-regulating the expression of these enzymes, it exacerbates oxidative stress (26).

### 2.3 Apoptosis-related miRNA

Alcohol consumption can significantly stimulate the expression of specific miRNA, which influence the cell life cycle by regulating their target genes. miR-200a, whose expression is upregulated in ALD, effectively inhibits the expression of ZEB2 mRNA by specifically binding to the 3'UTR of ZEB2 mRNA, which further leads to a decrease in the antiapoptotic protein Bcl-2 and an increase in the pro-apoptotic protein Bax increases, ultimately triggering the apoptotic process (27). miR-150-5p indirectly affects the ubiquitinated degradation of death domain proteins (FADDs) by inhibiting the expression of cytokine signaling inhibitor (CISH), induces exogenous apoptosis through the FADD/procaspase-8/caspase-dependent signaling cascade and further affects the apoptotic and survival status of hepatocytes (28). miR-34a maintains cellular homeostatic balance by finely regulating cell survival, proliferation, and cycling. miR-34a plays a key role in the response to cellular oxidative stress and DNA damage (29). However, overexpression of miR-34a inhibits the expression of its target gene SIRT1, which in turn triggers the acetylation process of P53 and P21, ultimately leading to apoptosis. Alcohol induces increased miR-34a expression, which further promotes apoptosis in hepatocytes and hepatic stellate cells through the SIRT1-p53 acetylation pathway (7, 30). These findings not only improve our understanding of the pathogenesis of ALD, but also provide new ideas and targets for the future diagnosis and treatment of ALD.

### 2.4 Inflammation-associated miRNA

The inflammatory response plays a central role in the development and progression of ALD. Several miRNA have been shown to influence this process by regulating key signaling pathways and gene expression. Chronic alcohol consumption led to the upregulation of miR-155 in hepatic Kupffer cells, which further led to the downregulation of negative regulators of the TLR4 pathway, such as IRAK-M and SOCS1, thereby attenuating the suppression of inflammatory responses. In addition, miR-155 exacerbated the inflammatory response by down- regulating the expression of histone deacetylase HDAC11 in Kupffer cells, which in turn promoted the expression of IL-10, enhanced the responsiveness of Kupffer cells to LPS, and increased the release of inflammatory factors such as TNF-*α* (31). Therefore, miR-155 knockdown not only attenuates alcohol-induced lipid accumulation by targeting PPAR-*α*, but also prevents alcohol- induced inflammation. One study found that miR-125a-5p reduced Kupffer cell tolerance to LPS by directly targeting TLR4, CD14 and effector cytokines (e.g., TNF-α, IL-6, etc.) (32). Significant down- regulation of miR-181b-3p in chronically alcohol-fed rats, which led to up-regulation of NFκB, a key transcription factor regulating inflammatory responses, exacerbated alcohol-induced inflammation. On the other hand, miR-21 promotes hepatic stellate cell activation and inflammatory responses through activation of the VHL/NF-κB pathway, which further releases inflammatory cytokines such as IL-6 and IL-1 *β*, further exacerbating the inflammatory state of hepatocytes (33). In addition, miR-182 is elevated in the liver ducts and hepatocytes of ALD patients, which directly affects the expression of inflammatory mediators (34).

### 2.5 Fibrosis-associated miRNA

In the pathological process of alcoholic liver injury, downregulation of miR-let-7a expression leads to an increase in Lin28B expression, which further promotes HSC activation and accelerates the progression of liver fibrosis (35). miR-148a-3p in hepatocytes inhibits ERBB3 expression, reduces cell viability, and promotes stellate cell apoptosis by regulating the signaling of its target gene ERBB3 (36, 37). On the other hand, in alcohol-treated rat livers, increasing the level of miR-148a-3p inhibited the expression of *α*-smooth muscle actin (α-SAM) and type I collagen, thereby ameliorating alcohol-induced liver fibrosis (38). In addition, miR-34a expression is upregulated in ALD, which contributes to the progression of liver fibrosis. In animal studies, inhibition of miR-34a suppressed TGF-β1/Smad2/3 signaling and attenuated liver fibrosis (39). The effect of alcohol on miRNA expression is also reflected in miR-19b and pri-miR-17-92. In primary hsc and LX2 cells, alcohol was able to down-regulate miR-19b expression and significantly up-regulate pri-miR-17-92, a change that further contributed to liver fibrosis (40). In conclusion, a variety of miRNA, including miR-let-7a, miR-let-7b, miR-148a-3p, miR-34a, and miR-19b, regulate the onset and progression of liver fibrosis through different molecular mechanisms, providing new potential targets for the treatment of liver fibrosis.

## 3 The role of lncRNA in ALD

**Figure 2:**
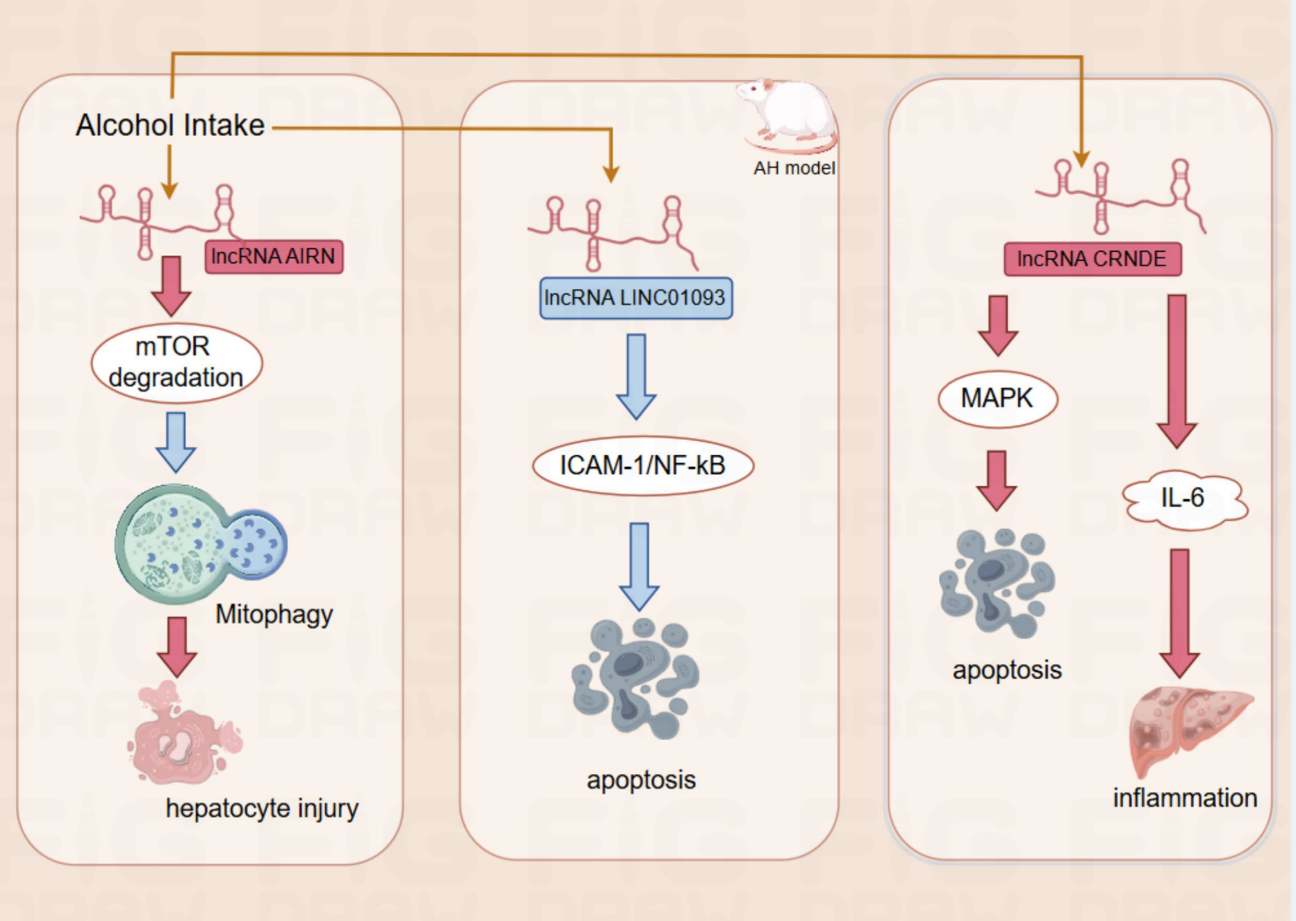
The red rectangles for upregulation, the blue rectangle for downregulation.The red arrows for promotion, the blue arrows for inhibition.AIRN by promoting mTOR degradation, it further suppresses mitophagy and eventually leads to hepatocyte injury; LINC01093 Inhibition of hepatocyte apoptosis by inhibiting the ICAM-1-mediated NF- *κ* B signaling pathway;CRNDE promotes apoptosis of hepatocytes by altering MAPK activity. Moreover, CRNDE also activates cytokines such as IL-6, which participate in the inflammatory response.

lncRNAs are closely associated with the development of liver disease and are not only involved in the regulation of various processes associated with liver injury, but also offer new possibilities for the diagnosis and treatment of ALD (Figure 2).

In ALD, the expression of the lncRNA AIRN is upregulated, which regulates the protein expression level of mTOR (mammalian target of rapamycin) by promoting its ubiquitination and degradation. mTOR is a key regulator of cell growth and metabolism, and reducing its activity contributes to promoting mitochondrial autophagy. Interfering with the expression of lncRNA AIRN can reduce the activity of mTOR, which in turn promotes mitochondrial autophagy and helps hepatocytes resist alcohol-induced injury (41). In addition, the expression of lncRNA LINC01093 was downregulated in an AH mouse model, resulting in its reduced inhibitory effect on the ICAM-1-mediated NF-κB signaling pathway, which further led to an increase in hepatocellular apoptosis (42). The lncRNA CRNDE was up-regulated in patients with ALD. CRNDE was directly involved in the development of ALD by altering the activity of specific signaling pathways such as MAPK (mitogen-activated protein kinase) activity, CRNDE directly regulates intracellular signaling in hepatocytes, thereby affecting the apoptotic process of hepatocytes. In addition, CRNDE activates cytokines such as IL-6, which are involved in the inflammatory response. These findings further demonstrate the important role of lncRNAs in the pathogenesis of ALD and provide a basis for their use as potential therapeutic targets and diagnostic markers (9). Validation in animal experiments and cellular models showed that the expression levels of lncRNAs AK054921 and AK128652 in the serum of ALD patients were closely associated with disease progression. Their expression levels were inversely correlated with the survival of patients with alcoholic cirrhosis, suggesting that these lncRNAs may serve as serum biomarkers to differentiate the progression of ALD and may also have predictive value for patient prognosis and survival (43). These findings not only improve our understanding of the pathogenesis of ALD but also provide new ideas and tools for clinical treatment and diagnosis. Although many studies have investigated the expression of lncRNAs in alcohol abuse-related diseases, little is known about their mechanism of action in ALD.

In ALD, the expression patterns of AIRN, LINC01093, and CRNDE in liver tissues do not show similar changes in other organs or diseases, suggesting that these lncRNAs may be liver-specific regulatory factors (9, 41, 42). This tissue-specific expression characteristic makes them potential biomarkers for liver diseases, particularly for early diagnosis of ALD. Monitoring the expression changes of AIRN, LINC01093, and CRNDE in liver tissue or serum could aid in the early detection of ALD and in tracking disease progression. Furthermore, the expression levels of AK054921 and AK128652 in the serum of ALD patients are closely associated with disease progression, and their changes are negatively correlated with the survival rate of patients with alcoholic liver cirrhosis (43). Therefore, they could serve as serum biomarkers to differentiate between various stages of ALD and provide valuable information for prognostic evaluation.

Thus, the tissue specificity of these lncRNAs not only makes them diagnostically relevant in ALD but also potentially offers new directions for targeted therapies for liver diseases. By modulating the expression of these lncRNAs, it may be possible to alleviate liver damage caused by ALD and improve clinical outcomes for patients.

## 4 Interaction and regulation of lncRNAs and miRNA

**Figure 3:**
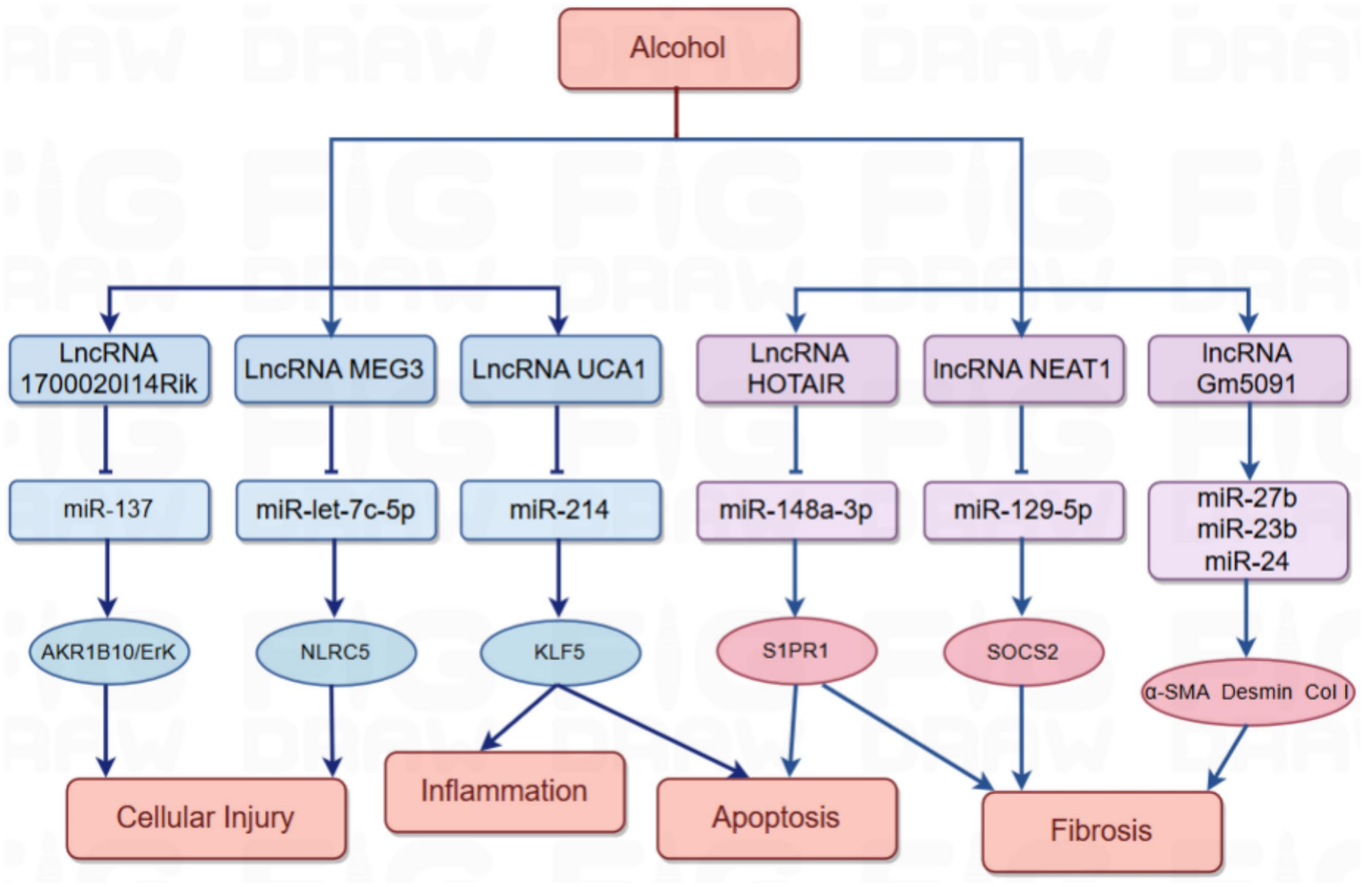
Interaction between LncRNAs and MiRNAs in alcoholic liver disease.

The synergistic effect of lncRNAs with microRNAs is more convincing in the study of ALD than the single effect of lncRNAs. With the development of high-throughput sequencing and other technologies, multiple interactions of lncRNAs with microRNAs are gradually being discovered, forming interconnected complex networks (Figure 3; Table 2).

**Table 2:** Regulatory mechanisms and function of the key lncRNA-miRNA axis in ALD.

lncRNA-miRNA	Regulatory mechanism	Functional impact	Reference
Gm5091/miR-27b/23b/24	Gm5091 adsorbs and reduces miR-27b/23b/24, inhibits HSC activation, and also significantly reduces IL-1β secretion and ROS production	Reduces fibrosis and oxidative stress	Wu et al. (44)
NEAT1/miR-129-5p	NEAT1 competes for binding to miR-129-5p, deregulates its inhibition of SOCS2, and enhances SOCS2-mediated anti-inflammatory signaling	Inhibition of inflammation and fibrosis	Zhou et al. (45)
UCA1/miR-214	UCA1 adsorbs miR-214, upregulates KLF5, promotes TNF-α, IL-6 release, and aggravates hepatocyte injury	Promotes inflammation and apoptosis	Luo et al. (47)
HOTAIR/miR-148a-3p	Inhibition of miR-148a-3p and up-regulation of S1PR1 promote HSC proliferation and migration	Promote fibrosis	Xiang et al. (48)
MEG3/miR-let-7c-5p	MEG3 competes with miR-let-7c-5p to promote NLRC5 expression and inhibit fatty acid oxidation and lipid scavenging.	Aggravate steatosis and apoptosis	Chen et al. (49)
AIRN/mTOR	Promote mTOR degradation and activate mitochondrial autophagy	Protect the hepatocytes from oxidative damage	Wan et al. (39)

In mouse liver tissue from AH model and alcohol-treated AML12 hepatocytes, lncRNA 1700020I14Rik indirectly increased the expression of AKR1B10 by inhibiting its activity through interaction with miR-137, which in turn activated the Erk pathway and enhanced the damage response of hepatocytes (44). In addition, alcohol-induced downregulation of the expression of lncRNA Gm5091 in hepatic stellate cells (HSCs). Gm5091 contains sites that bind to miR-27b, miR- 23b, and miR-24 and reduce the availability of miRNA by forming a complex with these miRNA, which reduces the expression of HSC activation markers *α*-SMA, desmin, and type I collagen expression. Overexpression of Gm5091 also significantly reduced secretion of the inflammatory factor IL-1β and intracellular ROS production, thereby attenuating hepatocyte inflammation, oxidative stress, and fibrosis (45). In the ASH mouse model, lncRNA NEAT1 slowed the liver fibrosis process in ASH by binding to miR-129-5p, reducing its availability in cells and further inhibiting its targeted inhibitory effect on the translation of SOCS2 mRNA. lncRNA NEAT1 is involved in the onset and progression of ALD by mediating the targeting of miR-129-5p to SOCS2 (46). The transcription factor KLF5 regulates the expression of pro-inflammatory cytokines (e.g., TNF-*α*, IL-1β, and IL-6) and affects hepatocyte survival and death by modulating the expression of apoptosis-related genes (e.g., Bcl-2 family proteins) (47). It was found that alcohol- induced upregulation of lncRNA UCA1. lncRNA UCA1 binds to miR-214 through its sequence, reduces the inhibitory effect of miR-214 on its target gene KLF5, promotes the expression of KLF5, and affects cellular inflammatory response and apoptosis. Knockdown of UCA1 expression can increase the level of miR-214, which in turn regulates the expression of KLF5, suggesting that the UCA1/miR-214/KLF5 axis plays a key role in alcohol-induced hepatocyte injury (48). In addition, it was found that the lncRNA HOTAIR can specifically bind to miR-148a-3p, regulate the expression of S1PR1 (sphingosine 1-phosphate receptor), and affect the proliferation and apoptosis of hepatic stellate cells. HOTAIR reduces the inhibitory effect of miR-148a-3p on its target S1PR1 by binding to miR-148a-3p, thereby upregulating S1PR1 expression, which promotes hepatic stellate cell activity, including proliferation and migration, and exacerbates liver injury and fibrosis. The important role of this axis in alcoholic liver injury has been demonstrated in the AH rat model by altering the phenotype of hepatic stellate cells through regulation of the HOTAIR/miR-148a-3p/S1PR1 axis (49). It was also found that the expression levels of lncRNA MEG3 and NLRC5 were significantly increased in an alcohol-fed model, whereas that of miR-let-7c-5p was significantly decreased. lncRNA MEG3, as a competing endogenous RNA (ceRNA) for miR-let-7c-5p, inhibited the interaction of miR-let-7c-5p with the binding of NLRC5’s 3' untranslated region (3'UTR), which promoted NLRC5 expression and further aggravated alcohol-induced steatosis and apoptosis (50).

In the future, the relationship between specific lncRNAs-miRNA needs to be studied in depth, and a deeper understanding of the synergistic effects of lncRNAs and microRNAs may provide new clues and significant discoveries for the treatment of ALD and other diseases.

## 5 Autopophagy and ER stress in ALD

Autophagy is a process by which cells maintain intracellular homeostasis through the degradation of damaged organelles and proteins (51). It plays a crucial role in the pathogenesis of ALD. Alcohol disrupts hepatic lipid homeostasis through multiple pathways, including promoting lipid synthesis, reducing lipoprotein secretion, and inhibiting fatty acid oxidation. Lipophagy, a specialized form of autophagy, is responsible for the degradation of lipid droplets in hepatocytes. Studies have shown that alcohol exposure can suppress lipophagy, leading to hepatic steatosis (52). Dynamin2 (DYN2) and GTPase RAB7 are critical for lipid droplet degradation and endosome-lysosome transport. Chronic alcohol consumption reduces their activity, thereby inhibiting lipophagy and promoting lipid accumulation (8, 10, 53, 54). Furthermore, alcohol decreases the number and functionality of hepatic lysosomes, impairing autophagic flux. TFEB, a master regulator of lysosomal biogenesis, plays a key role in this process. Acute alcohol intake increases nuclear TFEB levels in hepatocytes, whereas chronic alcohol exposure reduces its nuclear localization. TFEB knockdown exacerbates alcohol-induced liver damage, while its overexpression alleviates ALD-related pathological changes (55, 56). Chronic alcohol consumption activates the mTOR pathway, inhibiting TFEB nuclear translocation and thereby reducing autophagic activity. However, treatment with mTOR inhibitors such as Torin-1 can restore TFEB activity and mitigate liver damage (57, 58). Finally, chaperone-mediated autophagy (CMA) also plays a significant role in ALD. Studies have demonstrated that SNX10-deficient mice exhibit resistance to alcohol-induced liver damage. The absence of SNX10 upregulates LAMP2A, enhances CMA activity, and alleviates liver injury via the NRF2 and AMPK signaling pathways (59). Additionally, LCN2 deficiency sustains CMA activity and reduces liver damage caused by chronic alcohol consumption (60). These findings indicate that autophagy, particularly lipophagy, TFEB regulation, and CMA, plays a vital role in the progression of ALD and provides potential therapeutic targets for future interventions.

Alcoholic fatty liver disease (AFLD) is the primary manifestation of ALD, and its pathogenesis involves liver toxicity induced by ethanol and its metabolites, leading to oxidative stress, endoplasmic reticulum (ER) stress, and mitochondrial dysfunction. These processes trigger hepatocellular damage, inflammation, and liver fibrosis. ER stress occurs due to the accumulation of misfolded or unfolded proteins in the ER, resulting in ER dysfunction. The cell responds to this stress by activating the unfolded protein response (UPR) (61). ER stress is the trigger for UPR, which is a protective response by the cell to cope with ER stress. Studies have found that alcohol impairs the activity of methionine synthase, leading to hyperhomocysteinemia and ER stress, which further disrupts lipid metabolism and increases triglyceride and cholesterol production (62). Alcohol-induced UPR is typically transient, but chronic ALD leads to severe oxidative damage, inflammation, and cell apoptosis (63, 64), especially through the PERK-ATF6 signaling pathway, which upregulates NNMT and promotes lipid generation (65). In liver specimens from ALD patients, the ER protein Nogo-B is inhibited by CHOP signaling, which promotes M1 polarization of Kupffer cells and exacerbates liver damage (66). ER stress is influenced by factors such as acetaldehyde, hyperhomocysteinemia, and oxidative stress. The activation of UPR signaling in the later stages of ALD worsens lipid generation, inflammation, and apoptosis. Inhibiting UPR may offer potential therapeutic benefits for ALD treatment.

## 6 Exploring ncRNAs in non-alcohol dependence pathways

In addition to their roles in ALD, these ncRNAs are also widely involved in the regulation of various alcohol-independent diseases. Studies have shown that miR-155 promotes inflammatory signaling pathways in immune system diseases, such as rheumatoid arthritis (67), and in various cancers, such as breast cancer and lymphoma, by inhibiting tumor suppressor factors, thereby promoting cancer cell proliferation and metastasis (68). miR-223 plays a protective role in atherosclerosis and cardiovascular diseases by regulating inflammation and lipid metabolism (69), while in hematological diseases like leukemia, it exhibits dual roles, either promoting or suppressing cancer progression (70). As a downstream regulator of p53, miR-34a inhibits cell proliferation in various cancers, including ovarian and prostate cancers (71, 72), and impacts neuronal survival and synaptic plasticity in neurodegenerative diseases like Alzheimer’s disease (73). Furthermore, lncRNA HOTAIR has been widely demonstrated to promote cancer cell invasion and metastasis through chromatin remodeling in various malignancies, including lung, breast, and colorectal cancers (74), while also regulating inflammation and vascular remodeling in cardiovascular diseases (75, 76). These studies suggest that these ncRNAs not only play roles in ALD but are also involved in the key regulatory processes of various alcohol-independent diseases. Therefore, further investigation into their functions in different pathological contexts will help to more clearly distinguish their specific roles in ALD and provide new insights for the precision treatment of these diseases.

## 7 Perspective

The study of ncRNAs in ALD has provided insights into the pathogenesis of this complex disease at the molecular level and led to new directions for innovative diagnostic and therapeutic tools. However, specific, non-invasive, and more sensitive biomarkers for the diagnosis of ALD are still lacking.

Extracellular vesicles (EVs), including exosomes, transport a variety of bioactive molecules. As mediators of cell to cell communication of functional RNA (77, 78), these exosome-derived miRNA play important roles in disease initiation, development and resistance mechanism (79). The membrane structure of the exosome provides a natural protective barrier for miRNA, effectively preventing the degradation of enzymes and other chemicals, thus significantly improving miRNA stability (80, 81). In the field of hepatology, EVs has recently become a new player in the onset and progression of ALD (82–84). It has been shown that stressed hepatocytes promote exosome release and increase their content in specific proteins, lipids, and miRNA. These proteins, lipids, and miRNA modulate the transcriptional programs of adjacent hepatocytes and non-parenchymal cells (e. g., hepatic stellate cells, endothelial cells, cholangiocytes, Kupffer cells, and hepatic endothelial cells) (85). Thus regulating inflammation and fibrosis. EVs can also be secreted from other cells and affect liver inflammation. It has been shown that, On ethanol induction, the increased release of EVs from monocytes, and by delivering miR-27a with M2 polarization, stimulation of adjacent naive monocytes to polarization into M2 macrophages with increased IL-10 and TGF *β* secretion, thus contributing to the resolution of inflammation (86). Another study using an alcohol gastric infusion model developing ALD found that the let7f, miR-29a, and miR-340 were enriched in Evs from mouse blood, possibly as barcodes identifying ASH (87). EVs have been shown to be associated with the diagnosis and prognosis of AH. A study in trauma patients showed that only in the circulating EVs obtained by drinkers with evidence of liver injury, its specific miR barcode (miR-122 and let7f), and systemic inflammation-related markers IL-6 and IL-33 (88). This suggests that these miRNA can be used to detect “high-risk” populations for ALD. However, in ALD and other fields, its specificity and practical effect still need further study and verification.

Methylation modification is not only an important regulator of ncRNA expression and function, but ncRNAs can also influence methylation status (89), and RNA-based therapeutic strategies show great potential. For example, significant hepatic lipid accumulation is observed in a mouse model in which the hepatocyte histone methyltransferase Setdb1 is knocked out. In this process, upregulated miR-216b-5p binds to the 3'UTR of Setdb1 mRNA and interferes with its mRNA stability, which in turn exacerbates hepatic steatosis (90). Therefore, targeting strategies against hepatic Setdb1 may provide new ideas for the diagnosis and treatment of ALD. However, given the broad role of miRNA in many biological processes, their potential off-target effects need to be assessed comprehensively and cautiously.

In recent years, ncRNA-targeted therapeutic studies for ALD have gradually increased, but are still in the exploratory stage. And there are many studies using ncRNAs to regulate related liver diseases (e.g., viral hepatitis, non-alcoholic fatty liver disease (NAFLD), and hepatocellular carcinoma) at present. For example, miR-34a is a miRNA that is upregulated in ALD and other liver diseases and is involved in apoptosis and inflammatory responses. One study evaluated the safety and efficacy of a miR-34a mimic (MRX34) in patients with advanced solid-state tumors. Although this study does not directly target ALD, it provides preliminary data on miR-34a in clinical care.The findings show that MRX34 regulates apoptosis-related pathways while treating tumors, which provides important information on miR-34a as a therapeutic target for ALD (91). Another study found that in hepatitis C (HCV), miR-122 inhibitor (Miravirsen) was effective in reducing HCV viral load and improving liver function with good tolerability and safety. This is a miRNA therapy for HCV. However, this treatment also provides a theoretical basis for miR-122 therapeutic regimens in other liver diseases (e.g., ALD) (92). MALAT1 inhibition effectively slows down the course of NAFLD and beneficially affects the inflammatory response in the liver (93). Although this study focused on NAFLD, it demonstrates the potential of targeting the lncRNA MALAT1 in the treatment of liver disease, which also provides a potential therapeutic strategy for future applications in ALD.

The results of these clinical trials provide practical clinical data for the utilization of ncRNAs to modulate liver disease, and although most of the studies were not directly targeted at ALD, they provide valuable clinical experience and data support for the application of miRNA and lncRNAs in the treatment of ALD.

## 8 Summary

The study of lncRNAs and miRNA has deeply revealed the molecular biological mechanism of ALD, covering key biological pathways such as lipid metabolism, oxidative stress, inflammatory response, apoptosis and fibrosis, which not only helps to fully understand the development of ALD, but also helps in the early diagnosis and treatment of diseases.

However, there are still more limitations. First, the expression of many lncRNAs and miRNA is significantly affected by the stage of the disease and external environmental factors (e.g., amount of alcohol consumed, duration of alcohol consumption, etc.).This dynamic change may lead to instability of the biomarker, limiting its widespread clinical use. Second, because many lncRNAs and miRNA have similar expression patterns in other liver diseases (e.g., nonalcoholic steatohepatopathy, hepatocellular carcinoma), it reduces their specificity in the diagnosis of ALD and increases the risk of misdiagnosis. Finally, studies on lncRNAs and miRNA in ALD are relatively few and mostly focused on laboratory and animal models, with limited clinical sample size and lack of support from large-scale multicenter studies, which affects the generalizability and reproducibility of the findings.

Although lncRNAs and miRNA studies in ALD provide promising insights into disease mechanisms, diagnostic potential and therapeutic strategies, deeper studies are needed to overcome challenges such as specificity, delivery systems and large-scale validation to be used for future clinical applications.

## Glossary

ALD: Alcoholic liver disease
AH: Alcoholic hepatitis
AFL: Alcoholic fatty liver
lncRNAs: Long non-coding RNAs
miRNA: MicroRNAs
mTOR: Mammalian target of rapamycin
MAPK: Mitogen-activated protein kinase
HSCs: Hepatic stellate cells
GSR: Glutathione reductase
S1PR1: Sphingosine 1-phosphate receptor
CAMKK2: Calcium/calmodulin-dependent protein kinase kinase 2
AMPK: AMP-activated protein kinase
AKR1B10: Aldo-keto reductase family 1 member B10
**SREBP-1**: Sterol regulatory element-binding protein 1
S1PR1: Sphingosine-1-phosphate receptor 1
**HNF-1B**: Hepatocyte nuclear factor 1 beta
ROS: Reactive oxygen species
PIAS1: Protein inhibitor of activated STAT 1
PRMT1: Protein arginine methyltransferase 1
TLR: Toll-like receptor
TGF: Transforming growth factor
ERBB3: Epidermal growth factor receptor 3
ErK: Extracellular signal-regulated kinase
NLRC5: NOD-like receptor family CARD domain containing 5
KLF5: Kruppel-like factor 5
SOCS2: Suppressor of cytokine signaling 2
***α*-SMA**: Alpha-smooth muscle actin
EVs: Extracellular vesicles
EMT: Epithelial-mesenchymal transition
ACC: Coenzyme A carboxylase
PPAR*γ*: Peroxisome proliferator-activated receptor γ
HNF1B: HNF1 homology box B
Apo: Oapolipoprotein O
ACC1: Acetyl CoA carboxylase
FABP4: Fatty acid binding protein
LDLR: Low-density lipoprotein receptor-associated protein
LXR: Liver X-activated receptor
3'UTR: 3' untranslated region
GSR: Glutathione reductase
CISH: Cytokine signaling inhibitor
